# Low *Bifidobacterium* Abundance in the Lower Gut Microbiota Is Associated With *Helicobacter pylori*-Related Gastric Ulcer and Gastric Cancer

**DOI:** 10.3389/fmicb.2021.631140

**Published:** 2021-02-26

**Authors:** T. Barani Devi, Krishnadas Devadas, Meekha George, A. Gandhimathi, Deepak Chouhan, R. J. Retnakumar, Sneha Mary Alexander, Jijo Varghese, Sanjai Dharmaseelan, Sivakumar Krishnankutty Chandrika, V. T. Jissa, Bhabatosh Das, G. Balakrish Nair, Santanu Chattopadhyay

**Affiliations:** ^1^Rajiv Gandhi Centre for Biotechnology, Trivandrum, India; ^2^Government Medical College, Trivandrum, India; ^3^Genotypic Technology Pvt Ltd., Bengaluru, India; ^4^Manipal Academy of Higher Education, Manipal, India; ^5^Achutha Menon Centre for Health Science Studies, Sree Chitra Tirunal Institute for Medical Sciences and Technology, Trivandrum, India; ^6^Translational Health Science and Technology Institute, Faridabad, India

**Keywords:** *H. pylori*, gastric ulcer and cancer, gut microbiome, *Bifidobacterium*, probiotic

## Abstract

*Helicobacter pylori* infection in stomach leads to gastric cancer, gastric ulcer, and duodenal ulcer. More than 1 million people die each year due to these diseases, but why most *H. pylori*-infected individuals remain asymptomatic while a certain proportion develops such severe gastric diseases remained an enigma. Several studies indicated that gastric and intestinal microbiota may play a critical role in the development of the *H. pylori*-associated diseases. However, no specific microbe in the gastric or intestinal microbiota has been clearly linked to *H. pylori* infection and related gastric diseases. Here, we studied *H. pylori* infection, its virulence genes, the intestinal microbiota, and the clinical status of Trivandrum residents (*N* = 375) in southwestern India by standard *H. pylori* culture, PCR genotype, Sanger sequencing, and microbiome analyses using Illumina Miseq and Nanopore GridION. Our analyses revealed that gastric colonization by virulent *H. pylori* strains (*vacAs1i1m1cagA*+) is necessary but not sufficient for developing these diseases. Conversely, distinct microbial pools exist in the lower gut of the *H. pylori*-infected vs. *H. pylori*-non-infected individuals. *Bifidobacterium* (belonging to the phylum Actinobacteria) and *Bacteroides* (belonging to the phylum Bacteroidetes) were present in lower relative abundance for the *H. pylori*+ group than the *H. pylori*- group (*p* < 0.05). On the contrary, for the *H. pylori*+ group, genus *Dialister* (bacteria belonging to the phylum Firmicutes) and genus *Prevotella* (bacteria belonging to the phylum Bacteroidetes) were present in higher abundance compared to the *H. pylori-* group (*p* < 0.05). Notably, those who carried *H. pylori* in the stomach and had developed aggressive gastric diseases also had extremely low relative abundance (*p* < 0.05) of several *Bifidobacterium* species (e.g., *B. adolescentis*, *B. longum*) in the lower gut suggesting a protective role of *Bifidobacterium*. Our results show the link between lower gastrointestinal microbes and upper gastrointestinal diseases. Moreover, the results are important for developing effective probiotic and early prognosis of severe gastric diseases.

## Introduction

One of the most intriguing fundamental challenges in infectious disease research is to understand the combination of “microbial factors” and “other factors” that collectively determine clinical outcomes. For almost all bacterial infections, the pathogenic bacteria themselves and their virulence genes are extensively investigated, but the related “other factors” are often less studied or even ignored.

The gastric pathogen *Helicobacter pylori* is the main causative agent for gastric cancer (782,685 deaths/year) and gastric and duodenal ulcers (246,700 deaths/year), which together takes more than 1 million lives per year (GBD 2016 Causes of Death Collaborators, 2017; Bray et al., 2018). It is well known that the *vacuolating cytotoxin A (vacA)* and the *cytotoxin-associated gene A (cagA)* are the most critical bacterial genes that contribute to clinical outcomes (Isomoto et al., 2010; Hatakeyama, 2014). Both genes have polymorphic allelic structures and encode multitasking toxins. The VacA is a secreted toxin that penetrates gastric epithelial cells, produces large acidic vacuoles, and promotes cell death by stimulating intrinsic and extrinsic pathways of apoptosis, necrosis, and autophagy (Isomoto et al., 2010). In contrast, the CagA protein is injected by the bacterium into cells, where it interferes with actin cytoskeleton and tight junctions, and subverts pathways that regulate cell cycles (Hatakeyama, 2014). The role of these two toxins in gastric cancer and peptic ulcer were evaluated and confirmed by *in vitro*, *in vivo*, and clinical studies (Malfertheiner et al., 2014). For *vacA*, the *s1*, *i1*, and *m1* alleles encode the signal sequence, intermediate and mid regions of the protein, respectively, and these alleles are considered more toxigenic than the alternative *s2*, *i2*, and *m2* alleles (Atherton et al., 1995; Rhead et al., 2007; Basso et al., 2008; Chung et al., 2010). For *cagA*, the alleles that encode East-Asian CagA with “D” type-segments flanking its tyrosine phosphorylation motif EPIYA, rather than the alleles that encode Western type or “C” type-segments, are associated with more aggressive clinical outcomes (Isomoto et al., 2010; Hatakeyama, 2014). That said, however, ∼80–90% of the *H. pylori*-infected people, including those with the most virulent *vacA* and *cagA* alleles, do not develop any symptoms, while ∼10–20% suffer from different gastric diseases (Covacci et al., 1999). This implies that additional factors must contribute to the risks of overt diseases (Dorer et al., 2009).

The “other factors” that possibly play critical roles in determining the clinical outcomes are geography, host genetics, lifestyle, and gastrointestinal microbiota (Noto and Peek, 2017). Appreciating that the stomach harbors many bacteria other than *H. pylori*, and some of them may have a role in gastric diseases, the gastric microbiota was under intense study in the past decade (Bik et al., 2006; Li et al., 2009; Jo et al., 2016; Noto and Peek, 2017). Irrespective of the geographical locations, it was found that *H. pylori* colonization is associated with alteration of gastric microbiota, which is demonstrated by decreased microbial diversity and increased relative abundance of the bacteria under the phylum Proteobacteria, but the significance of these alterations are unknown (Andersson et al., 2008; Maldonado-Contreras et al., 2011; Das et al., 2017; Noto and Peek, 2017). Surprisingly, although the influences of intestinal microbiota in many non-communicable diseases including several cancers (e.g., colorectal cancer) are well-appreciated, not much attention was paid until recently to understand its significance in relation to *H. pylori* infection and associated gastric cancer (Saus et al., 2019). In murine model, it was shown that *H. pylori* colonization has distal effects including modulation in intestinal microbiota (Kienesberger et al., 2016). In human, *H. pylori* colonization in stomach was found to be associated with decreased abundance of the bacteria under the phylum Bacteroidetes as well as increased abundance of the bacteria under the phylum Proteobacteria and Firmicutes in intestine (Gao et al., 2018). However, no specific microbial species in the intestine has been shown to have a link with *H. pylori* infection and associated gastric diseases. This lack of information was the impetus for us to study the interrelations among the gastric *H. pylori* and its genotypes, the intestinal microbiota and the clinical status of hosts.

## Materials and Methods

### Study Population

Patients included in this study were having various upper gastrointestinal symptoms and seeking care at the Department of Gastroenterology, Government Medical College, Trivandrum (TMC). Trivandrum is the capital of Indian state Kerala, which is located in extreme South-West part of the country (Menon, 2017). The entire state including Trivandrum has Arabian Sea to the West and Western Ghats Mountain to the East. It is suggested that humans lived in this geographical region during Neolithic Age (Menon, 2000, 2017). Later, peopling of Kerala happened during 2–3 AD through land and sea. The modern Keralite community has diverse (Negroid, Proto-Australoid, Dravidian, and Aryan) lineages (Menon, 2017).

### Collection of Biological Materials

Two gastric biopsies were collected during upper GI endoscopy. One of them was taken in 600 μl of autoclaved Brucella broth containing glycerol and the other in 200 μl of phosphate-buffered saline (PBS; 0.22 μm membrane filtered; autoclaved). A stool sample was also collected. The biopsy and stool samples were transported to the Microbiome Laboratory of Rajiv Gandhi Centre for Biotechnology (RGCB) at 4°C and were stored immediately in a −80°C freezer until further processing. Written informed consents from patients were taken. The study was approved by the Institutional Human Ethical Committee of TMC (approval number: 05/07/2016/MCT) and RGCB (approval number: IHEC/01/2017/18).

### Detection of *Helicobacter pylori* Infection

DNA was extracted from gastric biopsy as described elsewhere (Das et al., 2017). The DNA was diluted to 4 ng/μl, and 1 μl of DNA was used in 20 μl of reaction volume containing 10 μl of EmeraldAmp GT PCR Master Mix (TaKaRa) and 2 μl of forward and reverse primers specific for *H. pylori ureB* (Supplementary Figure S1). A patient is considered to have *H. pylori* infection if the collected gastric biopsy showed the presence of *H. pylori* either by *ureB* PCR or by culture (described below) or by both techniques (Table 1 and Supplementary Tables S1, S2).

**TABLE 1:** The incidence of different upper GI diseases and the prevalence of *H. pylori* infection within the study group (*N* = 375).

					95% confidence interval	
Disease	Total	*H. pylori*+ (number)	*H. pylori*+ (%)	Odds ratio	LCI	UCI
Gastric cancer*	23	8	34.78	2.92	1.05	8.08
Gastric ulcer*	22	4	18.18	1.21	0.36	4.10
Duodenal ulcer*	6	3	50	5.47	1.01	29.70
Gastritis	135	40	29.63	2.3	1.19	4.47
NUD	97	15	15.46	1		
GERD	92	13	14.13	0.90	0.40	2.01

Of the 375 patients, 83 (22.1%) were infected with H. pylori, and 51 (13.6%) have severe gastric diseases (shown with *) like gastric cancer or peptic ulcer (gastric and duodenal ulcers), while the rest of the 324 patients have relatively milder diseases like non-ulcer dyspepsia (NUD), gastritis, and gastroesophageal reflux disorder (GERD). Of the 83 H. pylori-infected patients, 15 (18.1%) have severe gastric diseases. When compared to NUD, duodenal ulcer, gastric cancer, and gastritis shows 5.47 times, 2.92 times, and 2.3 times higher odds of H. pylori positivity, respectively.

### *Helicobacter pylori* Culture

The biopsies collected in Brucella broth were used for isolating *H. pylori* strains on Brain Heart Infusion (BHI) agar (2%) containing Dent (Oxoid), 0.4% IsoVitaleX (BBL), and 7% sheep blood. The inoculated plates were incubated in microaerobic condition (10% CO_2_, 5% O_2_, and 85% N_2_) at 37°C. *H. pylori* colonies (one colony for each patient) were further propagated as pure culture. *H. pylori* was identified by typical translucent colony morphology, Gram staining, as well as biochemical tests like urease, catalase, and oxidase (Supplementary Figure S2).

### *Helicobacter pylori* Genotyping

Genomic DNA was extracted from *H. pylori* strains as described elsewhere (Berg et al., 1997). RAPD-PCR was carried out in 25 μl of reaction volume containing 2.5 μl of primer 1,281 (10 pmole), 4 mM MgCl_2_, and 1.5 U of rTaq polymerase (TaKaRa) using previously described conditions (Berg et al., 1997; Mukhopadhyay et al., 2000). The multiplex PCR for *vacA* and *cagA* was performed using a modified protocol with 10 pmol of VA1-F/VA1-R, 10 pmol of VAG-F/VAG-R and 25 pmol of cag5c-F/cag5c-R, and 10 μl EmeraldAmp GT PCR Master Mix in 20 μl of reaction mix (Chattopadhyay et al., 2004). Characterization of other alleles of *vacA* and *cagA* by PCR and sequencing was done by previously described methods (Mukhopadhyay et al., 2000; Rhead et al., 2007; Chattopadhyay et al., 2012). The nucleotide sequences of the primers are given in Supplementary Table S3. For phylogenic analyses, PhyML 3.0 maximum likelihood trees were generated using bootstrapped (100 iterations) following estimation of an evolutionary model using full_modeltest_bootstrap genetic workflow in ETE3 python package (Guindon et al., 2010; Huerta-Cepas et al., 2016). The generated Newick tree files were used with the phylogram package for R to plot the phylogenetic trees (Wilkinson and Davy, 2018).

### Metagenomic Analysis of 16S rRNA Gene

DNA was extracted from 200 mg of stool following a previously described protocol (Bag et al., 2016). For the preparation of metagenome library, 30 ng DNA was used to amplify the V3–V4 region of bacterial 16S rRNA genes for 26 cycles using KAPA HiFi HotStart PCR kit (KAPA Biosystems Inc., Boston, MA, United States) (Supplementary Table S3). The products were further amplified for 10 cycles by index PCR to add the Illumina sequencing barcoded adapters (Nextera XT v2 Index Kit, Illumina, United States). The products were sequenced using Illumina MiSeq following manufacturer’s protocol. The paired end V3–V4 reads (275 × 2) were demultiplexed using bcl2fastq, quality checked using FastQC, stitched using Fastq-join and analyzed using QIIME. The query sequences were clustered using UCLUST method against a curated chimera-free 16s rRNA database (Greengenes v.13.8). The taxonomies were assigned using RDP classifier to these clusters at ≥97% sequence similarity against the reference database. The generated BIOM file was used for further analysis and visualization. The box plot analysis was done by R.

### Whole Genome Metagenome Analysis

For whole genome metagenome sequencing, 300 ng of genomic DNA was used after end-repairing (NEBnext ultra II end repair kit, New England Biolabs, MA, United States) and cleaning up with 1× AmPure beads (BeckmannCoulter, United States). The DNA samples were barcoded (LongAmp Taq 2× New England Biolabs, MA, United States) and cleaned up with 1.6× AmPure beads (Beckmann-Coulter, United States). The end-repairing was performed using NEBnext (New England Biolabs, MA, United States) and adapter ligation was performed for 10 min using NEB blunt/TA ligase (New England Biolabs, MA, United States). Library mix was cleaned up using 0.6× Ampure beads and finally eluted in 15 μl of elution buffer. The processed DNA samples were sequenced on GridION X5 (Oxford Nanopore Technologies, Oxford, United Kingdom) using SpotON flow cell (R9.4) in a 48 h sequencing protocol on MinKNOW 2.1 v18.05.5. Nanopore raw reads (“*fast5*” format) were base called (“*fastq5*” format) and demultiplexed using Albacore v2.3.1. The reads were compared against NCBI nr database using the diamond tool. The diamond BLASTX alignments were further converted to MEGAN readable format by using the NCBI taxonomy to summarize and order the results. MEGAN GUI is then used to estimate and interactively explore the taxonomical content by checking the read assignment from phylum to species level classification.

### PCR for *Bifidobacterium* Species

PCR with primers specific for *Bifidobacterium* species (Supplementary Table S3) was done using stool metagenomic DNA in a 20 μl PCR reaction. Similarly, PCR with DNA extracted metagenomically from gastric biopsies was also performed. We also performed quantitative PCR (qPCR) in triplicate for the stool DNA as well as the gastric biopsy DNA using *Bifdobacterium* species-specific primers. The qPCR was performed with Thermo Power SYBR Green Master Mix using 200 nM primers and 50 ng DNA. Standard program with annealing temperature of 55°C in Applied Biosystems QuantStudio 7 instrument was used.

### Statistical Analysis

For analyzing *H. pylori* infection status, clinical status and sex of the individual Chi-squared test was performed using Intercooled Stata 14.1 software to test the significance of the association between variables. Binary logistic regression was used to estimate the odds ratios with 95% confidence intervals. For metagenomics analysis, the statistical significances among the patient groups were calculated using the Kruskal–Wallis test (Kruskal–Wallis, *p* < 0.05).

## Results

### Patient Population, Clinical Status, and Prevalence of *Helicobacter pylori* Infections

The study includes a total of 375 adult (male: 181; female: 194; average age: 48.5 years) residents of Trivandrum city and suburbs. As shown in Table 1, the prevalence of *H. pylori* infection is remarkably low (83 of 375; 22.1%) in Trivandrum. Within the *H. pylori*-infected group, the total prevalence of severe gastric diseases (15 of 83; 18.1%) like gastric cancer and peptic ulcer (duodenal and gastric ulcers) are similar to other geographic regions. However, the distributions of different diseases were noticeably different from the rest of the country. For example, it is known that for most Indian states that duodenal ulcer is the major clinical outcome and gastric cancers are relatively less prevalent (Supplementary Table S4; Dhakal and Dhakal, 2018). In contrast, for Trivandrum, although the prevalence of total gastric cancer (23/375 or 6.1%) and gastric ulcer (22 or 5.9%) are high, the prevalence of duodenal ulcer (6/375 or 1.6%) is low (Table 1 and Supplementary Table S4).

Of the 83 *H. pylori*-infected patients, 35 were male (42.2%) and 48 were female (57.8%) (Table 2). The prevalence of severe gastric diseases like gastric cancer and peptic ulcer are significantly more in males (Table 2). Among the *H. pylori*-infected males, 34.3% had severe disease types, while in the corresponding female population, it was only 6.25%. The observed association between sex and disease status is statistically significant (*p* = 0.001) (Table 2).

**TABLE 2:** The disease types of the *H. pylori* positive (*N* = 83), males (*N* = 35) and females (*N* = 48) in the study.

Sex	Disease type		Total
	Mild	Severe	
Female	45 (93.7%)	3 (6.3%)	48 (100%)
Male	23 (65.7%	12 (34.3%)	35 (100%)
Total	68 (81.9%)	15 (18.1%)	83 (100%)

*The association between disease type (Severe: Gastric cancer/Peptic ulcer; Mild: Gastritis/NUD/GERD) and gender in H. pylori-positive group is shown. Among the 83 H. pylori-positive cases, disease severity is high among males compared with females (34.3% vs. 6.25%). The observed association between sex and disease status is statistically significant (p = 0.001). The estimated odds ratio for disease severity among males is 7.82 (95% CI: 2.0–30.53), which is statistically significant. Among the H. pylori positives, males have a 7.8 times higher odds of detecting with severe disease compared with females.*

### Genotypes of *Helicobacter pylori* Strains Isolated From Trivandrum

**FIGURE 1:**
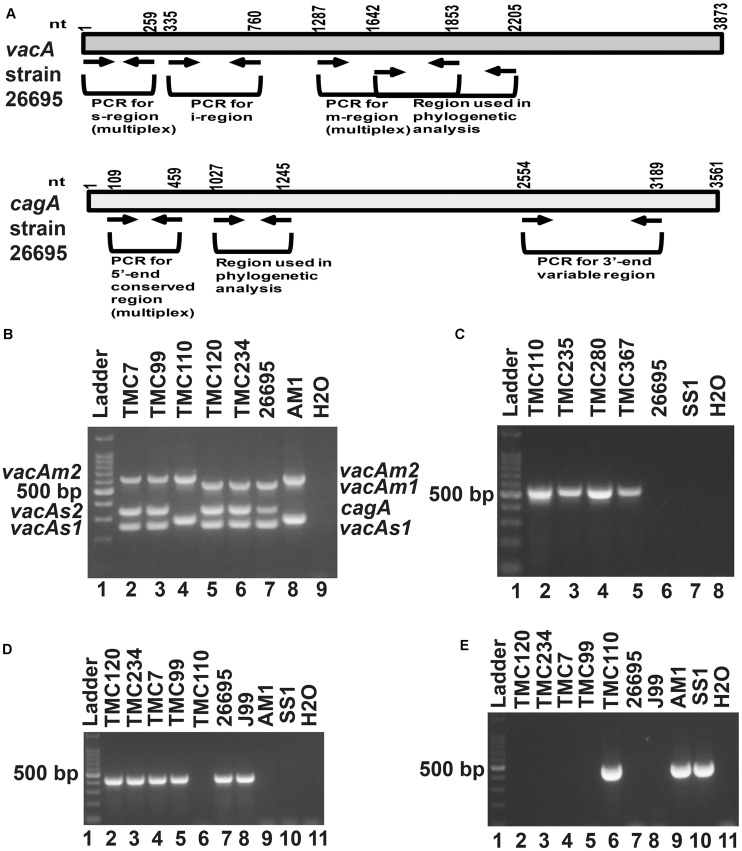
**(A)** Schematic diagram of the vacuolating cytotoxin A (*vacA)* and cytotoxin associated gene A (*cagA*) of the genome sequenced strain, 26,695 showing the nucleotide positions. The regions used in the analyses are indicated. **(B)** Multiplex PCR for genotyping the *vacAs1*, *vacAs2*, *vacAm1*, *vacAm2*, and *cagA* of *H. pylori*. The strains TMC7 and TMC99 (lane 2 and 3) have *vacAs1m2cagA*+; the strain TMC110 (lane 4) has *vacAs2m2cagA–*; the strains TMC120 and TMC234 (Lanes 5 and 6) have *vacAs1m1cagA*+ genotypes. The strains 26,695 (lane 7) and AM1 (lane 8) were used as positive controls for *vacAs1m1cagA*+ and *vacAs2m2cagA-* genotypes, respectively. Water (lane 9) was used as negative control. **(C)** The strains, TMC110, TMC235, TMC280, TMC367, which did not give amplicon for *cagA* in multiplex PCR, gave amplicon for the *cag*-empty site PCR. **(D)** PCR for the detection of *vacAi1* allele. The strains TMC120, TMC 234, TMC7, and TMC99 were found to carry the *vacAi1* allele, while the strain TMC110 did not give the amplicon. **(E)** PCR for the detection of *vacAi2* allele. The strain TMC110 was found to carry the *vacAi2* allele.

**FIGURE 2:**
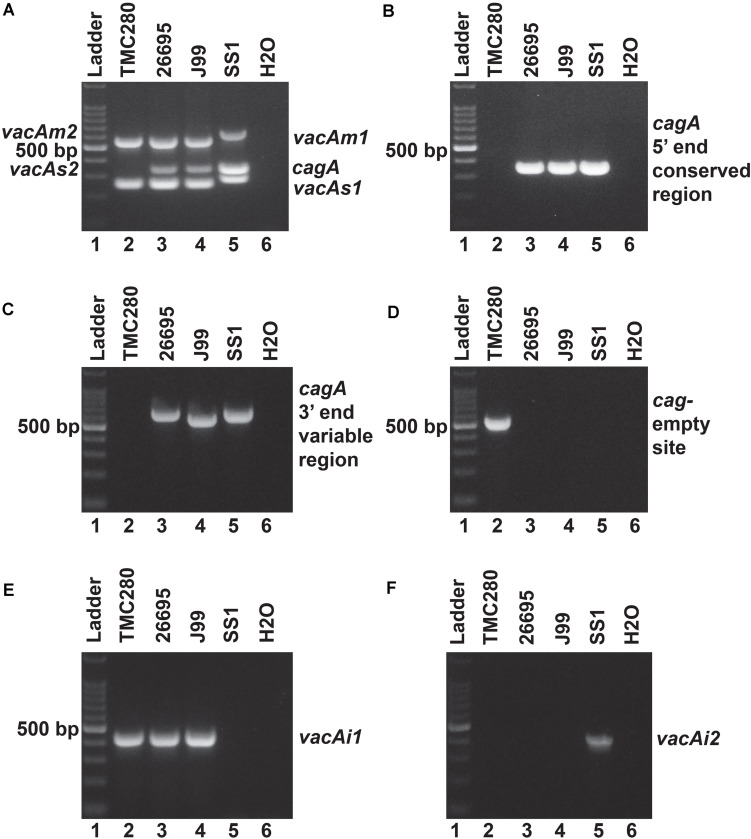
**(A)** Multiplex PCR for the characterization of *vacA* alleles and *cagA* gene of strain TMC280. The strain TMC280 carry *vacAs1m1cagA*- genotype, while the strains used as positive controls like 26,695 (*vacAs1m1cagA*+), J99 (*vacAs1m1cagA*+), and SS1 (*vacAs2m2cagA*–) gave amplicons at expected sizes. **(B)** No *cagA* specific amplicon were obtained for the strain TMC280 when the primers are targeted at the 5′ end conserved regions of the *cagA* gene. The positive controls, 26,695, J99, and SS, as expected, gave specific amplicons. **(C)** The PCR targeting the 3′ end variable region of the *cagA* gene. The strain TMC280 did not give any amplification with these primers, while the positive controls, 26,695, J99, and SS1, did. **(D)** The *cag*-empty site PCR. Only the strain TMC280, but not the strains 26,695, J99, and SS, gave amplicon for this PCR. **(E)** The PCR for the *vacAi1* allele. The strain TMC280, along with 26,695 and J99, gave amplicon for the *vacAi1* allele. The strain SS1, as expected did not give the *vacAi1* specific amplicon. **(F)** The PCR for the *vacAi2* allele. The strain TMC280 as well as the strains 26,695 and J99 were negative for this PCR, but the strain SS1 was positive. Therefore, it is confirmed that the strain TMC280 has *vacAs1i1m1cagA-* genotype.

**FIGURE 3:**
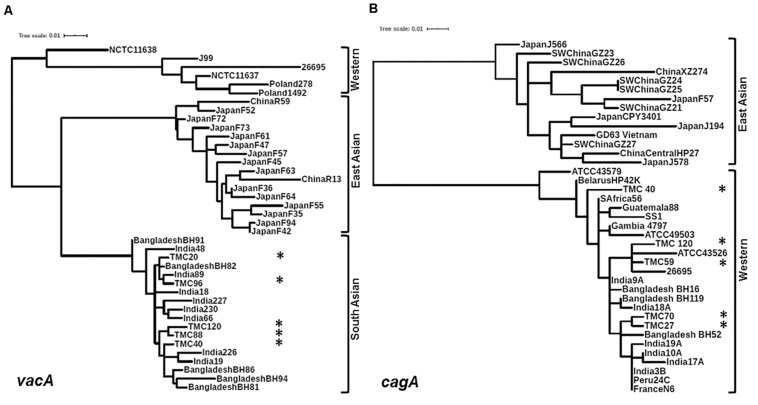
**(A)** Phylogenetic analysis of the *vacAm1*. The *vacAm1* of Trivandrum *H. pylori* strains (named as TMC and shown as asterisks) are closely related to the *vacAm1* of the *H. pylori* strains from South Asia, but differ from the *vacAm1* of *H. pylori* strains from Western world and East Asia. The *H. pylori vacAm1* sequences and their GenBank accession numbers are: TMC20 (MN968508); TMC40 (MN968509); TMC88 (MN968510); TMC96 (MN968511); TMC120 (MN968512); NCTC 11638 (HPU07145); Poland 1492 (AF097570); Poland 278 (AF097571); NCTC 11637 (AF049653); J99 (AE001511); 26695 (AE000598); Japan F73 (AF049652); Japan F72 (AF049651); Japan F52 (AF049631); China R59 (AF035611); Japan F61 (AF049645); Japan F57 (AF049634); Japan F35 (AF049625); Japan F55 (AF049632); Japan F47 (AF049629); Japan F45 (AF049628); Japan F94 (AF049640); Japan F42 (AF049626); Japan F36 (AF049642); Japan F64 (AF049647); Japan F63 (AF049635); China R13 (AF035610); Bangladesh BH91 (LC187447); India 48 (AF220112); India 18 (AF220110); India 227 (AF220116); India 230 (AF220117); India 66 (AF220113); India 89 (AF220114); Bangladesh BH82 (LC187444); Bangladesh BH94 (LC187448); India 226 (AF220115); India 19 (AF220111); Bangladesh BH81 (LC187443); Bangladesh BH86 (LC187446). **(B)** Phylogenetic analysis of the *cagA* 5′ end conserved region. The *cagA* of *H. pylori* strains isolated from Trivandrum (named as TMC and shown as asterisks) formed cluster within the *cagA* of Western *H. pylori* strains and not with the East Asian *H. pylori* strains. The *H. pylori cagA* sequences and their accession numbers are: TMC27 (MN968503); TMC40 (MN968504); TMC120 (MN968505); TMC59 (MN968506); TMC70 (MN968507); SW China GZ26 (KR154755); SW China GZ23 (KR154752); XZ274 China (NC_017926); Japan J566 (AB017922); SW China GZ27 (KR154756); GD63 Vietnam (CP031558); Japan J578 (AB017923); China Central HP27 (DQ306710); Japan CPY3401 (AY121840); Japan J194 (AB017921); Japan F57 (AB190935); SW China GZ21 (KR154750); SW China GZ24 (KR154753); SW China GZ25 (KR154754); ATCC 43579 (AB015414); HP42K Belarus (NZ_CP034314); India 17A (AF202223); S Africa 56 (AF198471); Guatemala 88 (AF198472); ATCC 49503 (AB015415); Gambia 4797 (AF198469); SS1 (KR154757); Bangladesh BH16 (LC187624); India 9A (AF202221); Bangladesh BH119 (LC187626); India 18A (AF202224); ATCC 43526 (AB015413); 26695 (AE000511); Bangladesh BH52 (LC187641); India 3B (AF202219); India 10A (AF202222); India 19A (AF202225); Peru 24C (AF198473); N6 France (CAHX01000001 to CAHX01000054).

Of the 83 *H. pylori* positive cases, 42 were positive by culture. DNA extracted from all 42 isolated *H. pylori* strains were used for genotyping the *vacA* signal sequence (*s*), mid (*m*), and intermediate (*i*) region alleles as well as the *cagA* 5′end conserved region and 3′end variable region. Of the 42 strains, 39 (92.9%) carried the toxigenic *vacAs1* allele, while three strains (7.1%) carried the non-toxigenic *vacAs2* allele (Figures 1A,B and Table 3). The prevalence of *vacAm1* (32 of 42; 76.2%) was higher than the prevalence of *vacAm2* (10 of 42; 23.8%). The *cagA* gene is present in 38 (90.5%) of 42 strains (Figures 1A,B and Table 3). Four strains (9.5%), which were negative for *cagA* gene, gave the 550 bp amplicon in *cag*-empty site PCR confirming that these four strains lacked the entire *cag*-PAI (Figure 1C and Table 3). The prevalence of *vacAi1* (37 of 42; 88.1%) allele was higher than the prevalence of *vacAi2* (5 of 42; 11.9%) allele (Figures 1D,E and Table 3). When the genotype data were combined, it was found that in Trivandrum, the *H. pylori* strains predominantly carry the most toxigenic *vacAs1i1m1cagA*+ genotype (73.8%), followed by the *vacAs1i1m2cagA*+ genotype (11.9%) (Table 3 and Supplementary Table S5). The strains that carry the less toxigenic *vacAs1i2m2cagA*+ (4.8%) and non-toxigenic *vacAs2i2m2cagA−* (7.1%) genotypes are relatively uncommon. One strain (TMC280) carried a rare *vacAs1i1m1cagA*− genotype (Figure 2, Table 3, and Supplementary Table S5). DNA fingerprinting analysis using randomly amplified polymorphic DNA (RAPD)-PCR for 11 representative strains of different genotypes showed unique pattern for each strain, suggesting that the Trivandrum *H. pylori* strains, like the *H. pylori* strains from other geographic regions, are highly diverse (Supplementary Figure S3). Phylogenetic analyses revealed that the *vacA* of Trivandrum *H. pylori* strains are related to the *vacA* of *H. pylori* strains isolated from South Asia (India, Bangladesh, etc.), while the *cagA* of the Trivandrum *H. pylori* strains formed cluster with the Western *cagA* (Figure 3).

**TABLE 3:** *H. pylori* genotypes and the clinical status of the host.

*H. pylori* genotypes		%	Clinical status											
Genotypes	No. of strains		Gastric Cancer (*n* = 3)		Gastric ulcer (*n* = 2)		Duodenal ulcer (*n* = 1)		Gastritis (*n* = 18)		NUD (*n* = 11)		GERD (*n* = 7)	
			No. of strains	%	No. of strains	%	No. of strains	%	No. of strains	%	No. of strains	%	No. of strains	%
*vacAs1*	39	92.8	3	100	2	100	1	100	17	94.4	10	90.9	6	85.7
*vacAs2*	3	7.1							1	5.6	1	9.1	1	14.3
*vacAm1*	32	76.2	1	33.3	1	50	1	100	15	83.3	9	81.8	5	71.4
*vacAm2*	10	23.8	2	66.7	1	50			3	16.7	2	18.2	2	28.6
*vacAi1*	37	88.1	2	66.7	1	50	1	100	17	94.4	10	90.9	6	85.7
*vacAi2*	5	11.9	1	33.3	1	50			1	5.6	1	9.1	1	14.3
*cagA(*+)	38	90.5	3	100	2	100	1	100	17	94.4	9	81.8	6	85.7
*cagA(−)*	4	9.5							1	5.6	2	18.2	1	14.3
*vacAs1i1m1cagA(*+)	31	73.8	1	33.3	1	50	1	100	15	83.3	8	72.7	5	71.4
*vacAs1i1m2cagA(*+)	5	11.9	1	33.3					2	11.1	1	9.1	1	14.3
*vacAs1i2m1cagA(*+)	0	0												
*vacAs1i2m2cagA(*+)	2	4.8	1	33.3	1	50								
*vacAs2i2m2cagA(−)*	3	7.1							1	5.6	1	9.1	1	14.3
*vacAs1i1m1cagA(−)*	1	2.4									1	9.1		

**FIGURE 4:**
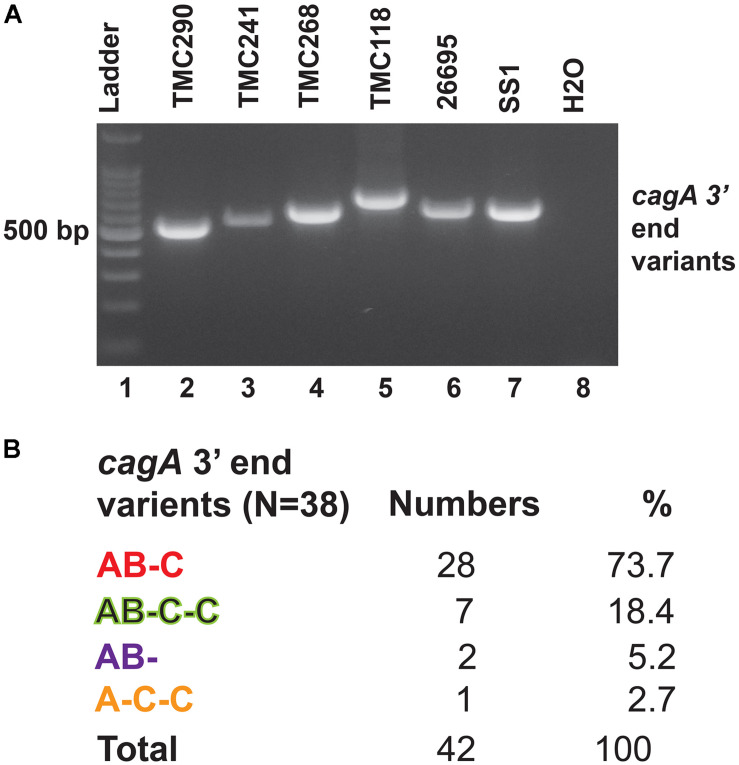
**(A)** PCR targeting the 3′ end variable regions of the *cagA* gene that encodes the EPIYA motifs. The amplicon sizes varied due to the variable number of EPIYA motifs and the spacer regions. **(B)** Different types of CagA types and their prevalence in Trivandrum. The nucleotide sequences (GenBank accession numbers MN968513 to MN968550) of the *cagA* genes were converted to the amino acid sequences and analyzed.

The 3′end variable region of the *cagA* gene that encodes variable numbers of EPIYA motifs and spacer sequences was studied by PCR and sequencing (Figure 4A). All *cagA* + *H. pylori* strains carried Western type-specific sequence (WSS) with C-segment (Figure 4B). Of the 38 *cagA* + strains, 28 (73.7%) were AB-C type with three EPIYA motifs, and seven (18.4%) were AB-C-C type with four EPIYA motifs. Two strains (5.2%) did not carry EPIYA motifs at the C segment, and they were AB- type with only two EPIYA motifs. One strain (2.7%) was found to have one EPIYA motif at the A site and two EPIYA motifs at the C sites, but no EPIYA motif at the B site, and therefore, this was the A-C-C type CagA. However, no association with the number of EPIYA motifs at the C-segment was found to have any clinical correlation (Supplementary Table S6).

Likewise, no association with clinical status of the host and genotypes of the *H. pylori* strains could be made (Table 3 and Supplementary Tables S6, S7). Therefore, the infection with putatively pathogenic types of *H. pylori* strains is dominant in all patient populations, but only certain people develop the severe gastric diseases like gastric cancer and peptic ulcer. These data suggest that, in addition to *H. pylori* infection and associated virulence genes, other factors contribute in determining clinical outcomes of the infected individuals.

### Gut Microbiota With Respect to *Helicobacter pylori* Infection and Clinical Status of the Host

**FIGURE 5:**
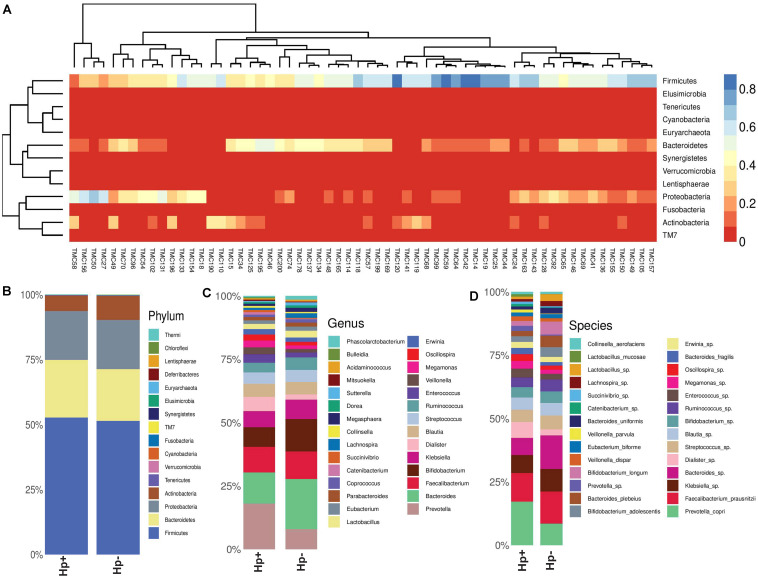
**(A)** Heat map showing the relative abundances of different phyla for each sample (*N* = 60). **(B)** Relative abundances of different phyla in gut shown in stacked column for the *H. pylori*+ (Hp+) and the *H. pylori*− (Hp−) groups. The Hp+ group has less relative abundance of the Phylum Actinobacteria. **(C)** Relative abundances of different genera in gut shown in stacked column for the *H. pylori*+ (Hp+; *N* = 30) and the *H. pylori*− (Hp−; *N* = 30) groups. The Hp+ group has less relative abundance of the genus *Bifidobacterium*. **(D)** Relative abundances of different bacterial species in gut shown in stacked column for the *H. pylori*+ (Hp+; *N* = 30) and the *H. pylori*− (Hp−; *N* = 30) groups. The Hp+ group has less relative abundance of the *Bifidobacterium longum* and *B. adolescentis.* The bacterial species that vary between the *H. pylori*+ and the *H. pylori−* groups are *B. longum* (Kruskal-Wallis, *p* = 0.009), *B. adolescentis* (*p* = 0.03), *B. bividum* (*p* = 0.004), *B. plebeius* (*p* = 0.05), *B. uniformis* (*p* = 0.04), and *P. copri* (*p* = 0.003).

As subsets of the study population, a total of 60 patients (30 *H. pylori*+ with average age 50.5 years; *H. pylori-* with average age of 50.4 years; same male to female ratio) were included in the gut microbiota analysis using Illumina MiSeq (275 × 2) platform (Supplementary Table S8). The rarefaction curve used as a measure of depth of sequencing is shown in Supplementary Figure S4. It is evident that for most patients, the abundant gut bacterial phyla is Firmicutes, followed by Bacteroidetes, Proteobacteria, Tenericutes, and Actinobacteria (Supplementary Figures S5, S6). However, for few patients like TMC27 (a patient with GERD; infected with *H. pylori*), TMC50 (a patient with gastritis; not infected with *H. pylori*), TMC58 (a patient with non-ulcer dyspepsia; not infected with *H. pylori*), TMC131 and TMC156 (two patients with gastritis; infected with *H. pylori*). Proteobacteria is the dominant phylum in the gut (Figure 5A and Supplementary Figure S6). No patient with severe gastric diseases like gastric cancer or peptic ulcer was found to have Proteobacteria as dominant phylum in the fecal microbiome. Overall, as shown in the heat map, four phyla, Firmicutes, Bacteroidetes, Proteobacteria, and Actinobacteria showed wide variation in abundance among individuals (Figure 5A). Principal Coordinates Analysis (PCoA) showed wide variations among subjects, but many *H. pylori*+ cases are found to be closely related to each other (Supplementary Figure S7). Likewise, many *H. pylori-* cases are also closely related to each other. This strongly suggests that the *H. pylori*+ group and the *H. pylori-* group may have respective unique components, which are distinct from the other group.

To this end, we looked into comparative analysis of the gut microbiota composition for the *H. pylori*+ (*N* = 30) and *H. pylori*− (*N* = 30) patients irrespective of clinical status of the host. Most of the *H. pylori*+ patients have more diverse gut microbiota than their respective age- and sex-matched *H. pylori-* counterparts (Supplementary Table S9). Collectively, the *H. pylori*+ group has more alpha diversity than the *H. pylori-* group as was discerned by the Shannon index, Simpson index, and Chao1 index analyses (Supplementary Figure S8). When the bacterial relative abundance was taken into account between two groups, it was noticed that the abundance of phylum Actinobacteria was lower and phylum TM7 (Saccharibacteria) was higher in the *H. pylori*+ group than in the *H. pylori-* group (Figure 5B). Similar analysis at the genus level showed that *Bifidobacterium* (belonging to the phylum Actinobacteria) and *Bacteroides* (belonging to the phylum Bacteroidetes) were present in lower relative abundance for the *H. pylori*+ group than the *H. pylori-* group (Figure 5C). Conversely, for the *H. pylori*+ group, genus *Dialister* (bacteria belonging to the phylum Firmicutes) and genus *Prevotella* (bacteria belonging to the phylum Bacteroidetes) were present in higher abundance as compared to the *H. pylori-* group (Figure 5C). The species of the *Bifidobacterium* that vary between the *H. pylori*+ and the *H. pylori-* groups are found to be *B. longum* (Kruskal–Wallis, *p* = 0.009), *B. adolescentis* (*p* = 0.03), and *B. bividum* (*p* = 0.004), although some of the species remained unidentified at this point of the analyses (Figure 5D). Similarly, for the *Bacteroides* and *Prevotella*, the species that could be identified by the 16S rRNA gene analyses are *B. plebeius* (*p* = 0.05), *B. uniformis* (*p* = 0.04), and *P. copri* (*p* = 0.003), respectively, while it was noticed that other species were also present but were not identifiable by V3–V4 regions of the 16S rRNA gene sequence analyses (Figure 5D). The species of the genus *Dialister* (*p* = 0.05) also could not be identified by this analysis (Figure 5D).

**FIGURE 6:**
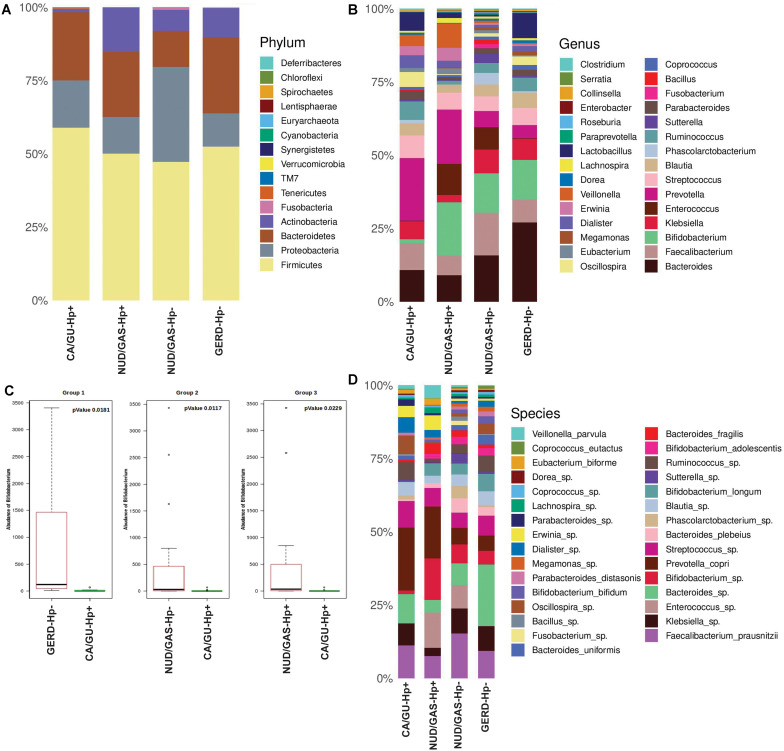
**(A)** Relative abundances of different phyla in gut shown in stacked column for the CA/GU-Hp+, NUD/GAS-Hp+, NUD/GAS− Hp−, and GERD-Hp− groups. The CA/GU-Hp+ group have low relative abundance of Actinobacteria as compared to the other groups. **(B)** Relative abundances of different genera in gut shown in stacked column plot for the CA/GU-Hp+, NUD/GAS-Hp+, NUD/GAS- Hp−, and GERD-Hp− groups. Relative abundance of *Bifidobacterium* for the CA/GU-Hp+ group is significantly low. **(C)** Box plot analysis showing that the abundance of *Bifidobacterium* is significantly lower in the CA/GU-Hp+ group than the NUD/GAS-Hp+, NUD/GAS- Hp−, and GERD-Hp− groups. **(D)** Relative abundances of different bacterial species in gut shown in stacked column for the CA/GU-Hp+, NUD/GAS-Hp+, NUD/GAS- Hp−, and GERD-Hp− groups. Significantly low relative abundances of *Bifidobacterium bifidum*, *Bifidobacterium adolescentis*, and *Bifidobacterium longum* are identified for the CA/GU-Hp+ group.

Since our analyses pointed out differences in the composition of the gut microbiota between the *H. pylori*+ and the *H. pylori-* patients, our next aim was to find the distinctiveness in the gut microbiota of the *H. pylori*+ patients with severe gastric disorders. Therefore, we compared *H. pylori*+ patients with gastric cancer or gastric ulcer (CA/GU-Hp+) with patients having milder clinical outcomes like non-ulcer dyspepsia or gastritis with *H. pylori* infection (NUD/GAS-Hp+), non-ulcer dyspepsia, or gastritis without *H. pylori* infection (NUD/GAS-Hp−) and gastroesophageal reflux disease without *H. pylori* infection (GERD-Hp−). The metadata related to these four patient groups are given in Supplementary Table S10. It was noticed that the CA/GU-Hp+ group has remarkably low relative abundance of bacteria belonging to the phylum Actinobacteria (Figure 6A). Further analyses revealed that the most significant uniqueness of the CA/GU-Hp+ patients is the low relative abundance of the genus *Bifidobacterium* (under the phylum Actinobacteria) (Figure 6B). The abundance of *Bifidobacterium* in the CA/GU-Hp+ group was found to be significantly lower than the GERD-Hp− (*p* = 0.0181), NUD/GAS-Hp− (*p* = 0.0117), and NUD/GAS-Hp+ (*p* = 0.0229) groups (Figure 6C). This finding was further confirmed by heat map (Supplementary Figure S9). Further species level analysis revealed that several *Bifidobacterium* species like *B. adolescentis* (*p* = 0.005 with respect to NUD/GAS-Hp−; *p* = 0.003 with respect to NUD/GAS-Hp+; *p* = 0.009 with respect to GERD-Hp−), *B. longum* (*p* = 0.002 with respect to NUD/GAS-Hp−; *p* = 0.008 with respect to NUD/GAS-Hp+; *p* = 0.02 with respect to GERD-Hp−), and *B. bifidum* (*p* = 0.01 with respect to NUD/GAS-Hp−; *p* = 0.01 with respect to GERD-Hp−) were present at significantly lower relative abundance specifically for the CA/GU-Hp+ group compared with the other groups (Figure 6D). The CA/GU-Hp+ group also have high *Oscillospira* (*p* = 0.007 with respect to NUD/GAS-Hp−; *p* = 0.002 with respect to NUD/GAS-Hp+; *p* = 0.02 with respect to GERD-Hp−). However, we noticed that 16S rRNA gene analyses were not able to identify all *Bifidobacterium* species that are present at high abundance in the gut of NUD/GAS-Hp+, NUD/GAS- Hp−, and GERD-Hp− patients but were present at a significantly lower abundance in the gut of CA/GU-Hp+ patients (Figure 6D).

The 16S rRNA gene analyses could not resolve all *Bifidobacterium* species. Therefore, we decided to identify them by whole genome metagenome sequencing. For this experiment, we have chosen the Oxford Nanopore technology in GridION X5 platform for its longer read length. A total of six patients were chosen. Three of them are CA/GU-Hp+ and three are NUD/GAS-Hp+. The samples are age and sex matched and all individuals are *H. pylori*+ to avoid any bias. Details of the metadata are given in Supplementary Table S11. The Nanopore read statistics for each sample is given in Supplementary Table S12. The abundance of each domain in each sample is shown in Supplementary Table S13. Our analysis has identified a total of eight *Bifidobacterium* species, which were remarkably different between the two groups and for each age- and sex-matched pairs (Table 4). Seven (*B. adolescentis*, *B. bifidum*, *B. breve*, *B. longum*, *B. moukalabense*, *B. pseudocatenulatum*, and *B. reuteri*) of the eight *Bifidobacterium* species were present at a very low abundance in the intestine of the CA/GU-Hp + individuals than the corresponding age- and sex-matched NUD/GAS-Hp+ individuals (Table 4). The lower abundance of the *Bifidobacterium* in CA/GU-Hp+ individuals compared with the NUD/GAS-Hp+ individuals is also confirmed with regular PCR (Supplementary Figure 10) and quantitative PCR (Supplementary Figure 11 and Supplementary Table S14).

**TABLE 4:** The read assignment to *Bifidobacterium* species in whole genome metagenome sequencing and analysis for three paired (age and sex matched) CA/GU-Hp+ vs. NUD/GAS-Hp+ subjects.

	CA/GU-Hp+	NUD/GAS-Hp+	CA/GU-Hp+	NUD/GAS-Hp+	CA/GU-Hp+	NUD/GAS-Hp+

Species	TMC99	TMC110	TMC163	TMC125	TMC154	TMC120
*Bifidobacterium adolescentis*	0	29	0	202	0	644
*Bifidobacterium bifidum*	0	47	0	0	0	37
*Bifidobacterium breve*	38	10,152	0	0	0	47
*Bifidobacterium dentium*	0	0	0	0	366	0
*Bifidobacterium longum*	0	1,078	0	325	27	1,350
*Bifidobacterium moukalabense*	0	0	0	0	39	0
*Bifidobacterium pseudocatenulatum*	0	0	0	74	0	0
*Bifidobacterium reuteri*	0	52	0	0	0	0
Total	38	11,358	0	601	432	2,078

## Discussion

Why most *H. pylori*-infected individuals remain asymptomatic while a certain proportion develops gastric ulcer, duodenal ulcer, and gastric cancer had remained enigmatic. Substantial literature has accumulated on the *H. pylori* virulence genes like *vacA* and *cagA* to understand their roles in pathogenesis, but relatively fewer attempts were made to understand the “other factors” that influence and determine clinical outcomes (Isomoto et al., 2010; Palframan et al., 2012; Hatakeyama, 2014). The other factors that are likely to be involved in determining the clinical status are polymorphism of several cytokine genes and lifestyle of the individuals, which includes diet, alcohol consumption, and smoking (Ladeiras-Lopes et al., 2008; Moy et al., 2010; Ren et al., 2012; Datta De and Roychoudhury, 2015).

It is hypothesized that the gastrointestinal microbiota also contributes to the clinical outcome. Dysbiosis of the gastrointestinal microbiota is associated with many communicable and non-communicable as well as chronic and acute diseases, and therefore, it is likely that it could also be involved in gastric cancer and peptic ulcer (Hsiao et al., 2014; Forslund et al., 2015; Rogers, 2015; Zhang X. et al., 2015; Bratburd et al., 2018; Saus et al., 2019). It is also not unlikely that a beneficial microbe in the gastrointestinal microbiota is involved in protecting certain individuals from *H. pylori*-mediated pathogenesis. A similar protective role of *Ruminococcus obeum* (later classified as *Blautia obeum*) in the human gut was found against the infection of intestinal pathogen *Vibrio cholerae* (Hsiao et al., 2014). It has been shown that *H. pylori* infection tends to decrease the overall microbial diversity in stomach with a preferential increase in the relative abundance of bacteria belonging to the phylum Proteobacteria, but no specific association between a particular microbial species in stomach and *H. pylori-*related gastric diseases was found (Andersson et al., 2008; Das et al., 2017). Fewer studies on intestinal microbiota in the context of *H. pylori* infection and gastric diseases also could not identify the same (Gao et al., 2018). The present study, which involved analyses of clinical data, *H. pylori* genotype data, and metagenomics data, revealed that gastric colonization of virulent *H. pylori* strain is necessary but not sufficient for developing severe gastric diseases. Rather, reduced abundance of several species of *Bifidobacterium* in intestinal microbiota (but not in the stomach microbiota) is linked to *H. pylori* infection and related severe gastric diseases (Figure 6 and Supplementary Table S14).

The bacteria belonging to the genus *Bifidobacterium* are one of the first colonizers in human gut after birth, and the overall health benefits (including anti-tumor immunity) of these lactose fermenting bacteria are well appreciated (Sivan et al., 2015; O’Callaghan and van Sinderen, 2016; Oki et al., 2018). Therefore, several clinical trials were conducted using *Bifidobacterium*, along with *Lactobacillus* or *Streptococcus* as probiotic supplements with antibiotics and proton pump inhibitors to eradicate *H. pylori* (Zhang M.M. et al., 2015; Wang et al., 2017). These studies showed moderate levels of improvements with *H. pylori* eradication and reductions of the side effects of therapy (Zhang M.M. et al., 2015; Wang et al., 2017). The abundance of *Bifidobacterium* in the gut was increased after a successful eradication of *H. pylori* (Guo et al., 2019). Likewise, gastric ulcers induced by acetic acid or ethanol in mice healed faster when the mice were colonized with *Bifidobacterium* (Nagaoka et al., 1994). Although the entire mechanism is not revealed, it is known that multiple mechanisms like modulation of NFkB signaling and synthesis of antimicrobial peptides are involved in the *Bifidobacterium-*mediated inhibition of *H. pylori* (Collado et al., 2005; Shirasawa et al., 2010). However, it was not known whether or not a real difference in the abundance of *Bifidobacterium* exists between the *H. pylori*-infected human with severe gastric diseases and the *H. pylori*-infected human without these diseases. Our data filled that gap by clearly showing that no virulence gene of *H. pylori* is associated with severe gastric diseases unless the relative abundance of *Bifidobacterium* in the lower gut is significantly low. To the best of our knowledge, this is the first study, which has identified a specific lower gut microbe is linked to the gastric disorders that take over a million lives every year. The lower abundance of the beneficial microbe *Bifidobacterium* in the lower gut may serve as non-invasive assessment of gastric cancer and gastric ulcer risks. Furthermore, some of the *Bifidobacterium* strains may also serve as effective probiotics against gastric cancer and gastric ulcer.

## Conclusion

Numerous *in vitro* and *in vivo* studies have convincingly proved the pathogenic potential of *H. pylori vacA* and *cagA*. However, molecular epidemiology data showed that only 10–20% of the *H. pylori*-infected individuals develop gastroduodenal diseases. Why 80–90% *H. pylori* infections remain benign is unknown. The present study involving *H. pylori vacA* and *cagA* genotypes and fecal microbiota analyses revealed that apart from the virulence genes of the *H. pylori* strains, the intestinal microbiota is also involved in the context of *H. pylori* infection and the related gastric diseases. We have identified several species of *Bifidobacterium* (phylum Actinobacteria) that are present at very low abundance specifically in the gut of *H. pylori*-infected patients with severe gastric diseases suggesting a protective role of this beneficial microbe against severe gastric diseases. This finding may lead to developing early prognosis of severe gastric diseases or developing probiotics conferring protection against these diseases.

## Data Availability Statement

The GenBank accession numbers of nucleotide sequences of *H. pylori* genes are as follows: the 3′ end variable regions of *cagA* genes are MN968513 to MN968550; the 5′ end regions of *cagA* genes are MN968503 to MN968507; the m1 alleles of vacA genes are MN968508 to MN968512. The NGS (Illumina MiSeq) dataset related to microbial 16S rRNA gene is available at https://www.ncbi.nlm.nih.gov/Traces/study/?acc=PRJNA605091. The NGS (GridION X5) data related to the whole genome metagenome sequencing is available at https://www.ncbi.nlm.nih.gov/Traces/study/?acc=PRJNA648297.

## Ethics Statement

The studies involving human participants were reviewed and approved by the Institutional Human Ethical Committee of RGCB. The patients/participants provided their written informed consent to participate in this study.

## Author Contributions

SC conceived the idea. TD, KD, MG, DC, RR, SA, JV, SD, and SC performed the experiments. TD, AG, SKC, VTJ, and SC analyzed the data. TD, KD, AG, RR, BD, GN, and SC wrote the manuscript. All authors contributed to the article and approved the submitted version.

## Conflict of Interest

AG was employed by the company Genotypic Technology Pvt. Ltd, Bengaluru, Karnataka, India. The NGS was outsourced to this company. Some of the analysis was done by AG upon request in the absence of any commercial or financial relationships. The remaining authors declare that the research was conducted in the absence of any commercial or financial relationships that could be construed as a potential conflict of interest.
