# Supplementary material for: Low Bifidobacterium Abundance in the Lower Gut Microbiota Is Associated With Helicobacter pylori-Related Gastric Ulcer and Gastric Cancer
Source: Front Microbiol. 2021 Feb 26;12:631140. doi: 10.3389/fmicb.2021.631140 (PMC7953064; doi:10.3389/fmicb.2021.631140)
Supplement: Supplementary file 1 [file Data_Sheet_1.pdf]

Supplementary Material

Supplementary Figures

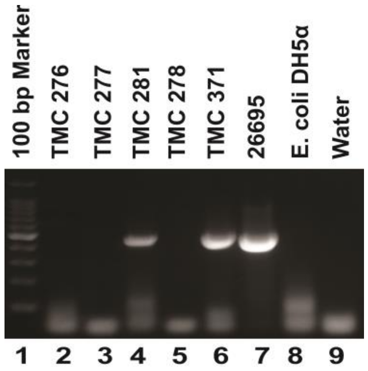

Figure S1. PCR for the amplification of the of *H. pylori ureB* gene that encodes the urease enzyme. The metagenomics DNA extracted from the gastric biopsies were used for the *ureB* PCR for the presence of *H. pylori* in biopsy samples.

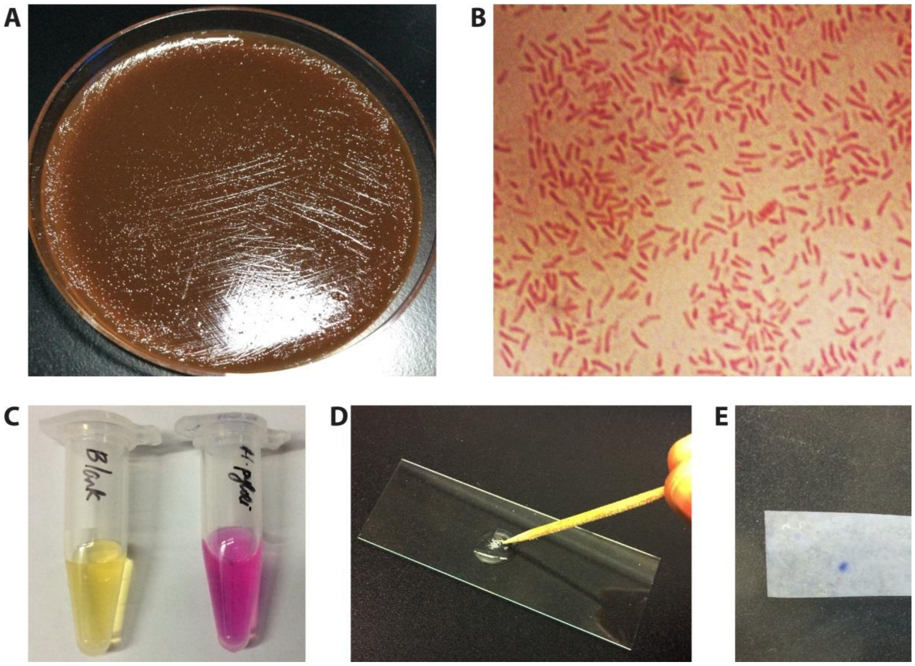

Figure S2. (A) *H. pylori* colonies grown on BHI-agar plate supplemented with sheep blood. The colonies look small and transparent like water droplets. (B) *H. pylori* visualized under microscope (1000X) after Gram staining. The Gram negative curved bacilli were visible. (C) Positive urease test (pink) for *H. pylori*. (D) Positive catalase test (bubbles) for *H. pylori*. (E) Positive oxidase test (purple color) for *H. pylori*.

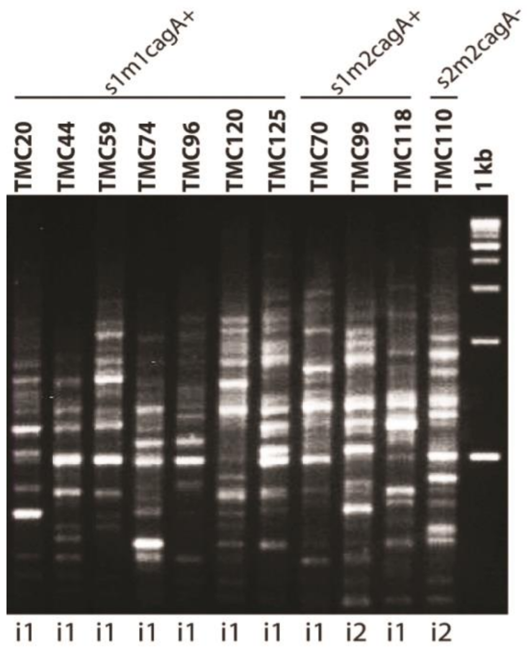

Figure S3. DNA fingerprinting analysis using randomly amplified polymorphic DNA (RAPD)-PCR for 11 representative *H. pylori* strains with different genotypes. Seven of them were *vacAs1i1m1cagA+*, 2 of them were *vacAs1i1m2cagA+*, 1 of them was *vacAs1i2m2cagA+* and 1 of them was *vacAs2m2cagA*-. Each strain has unique RAPD pattern.

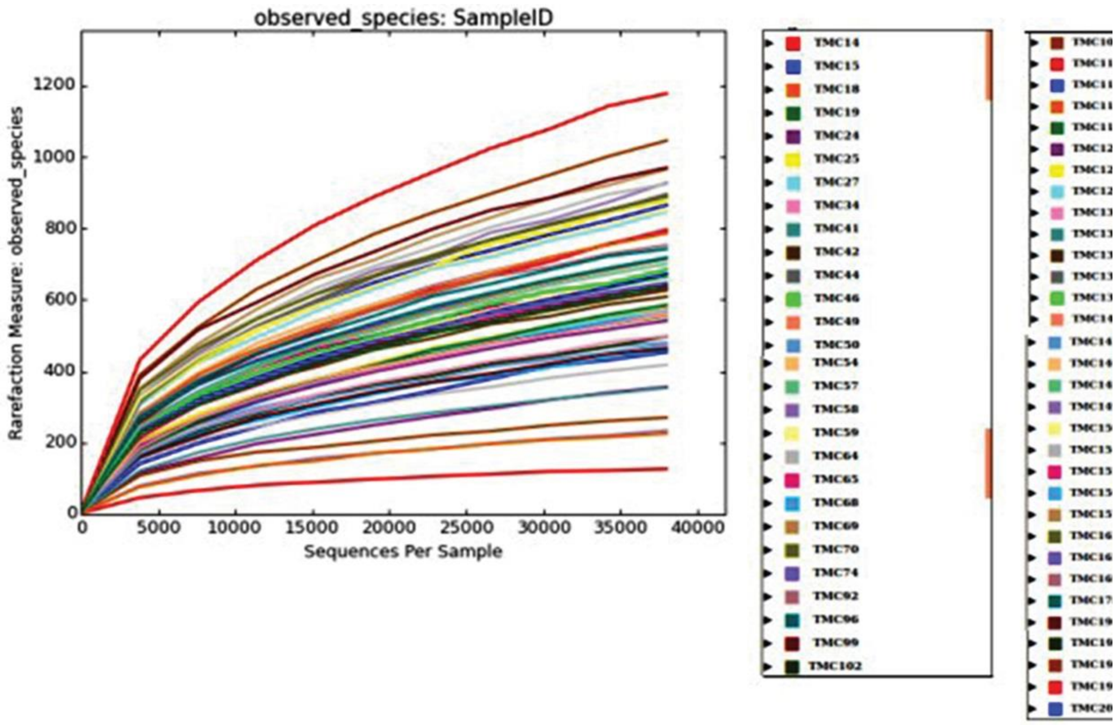

Figure S4. Rarefaction curve of the NGS data obtained from sequencing the V3-V4 region of the 16S rRNA genes of the bacteria present in the stool of Trivandrum residents.

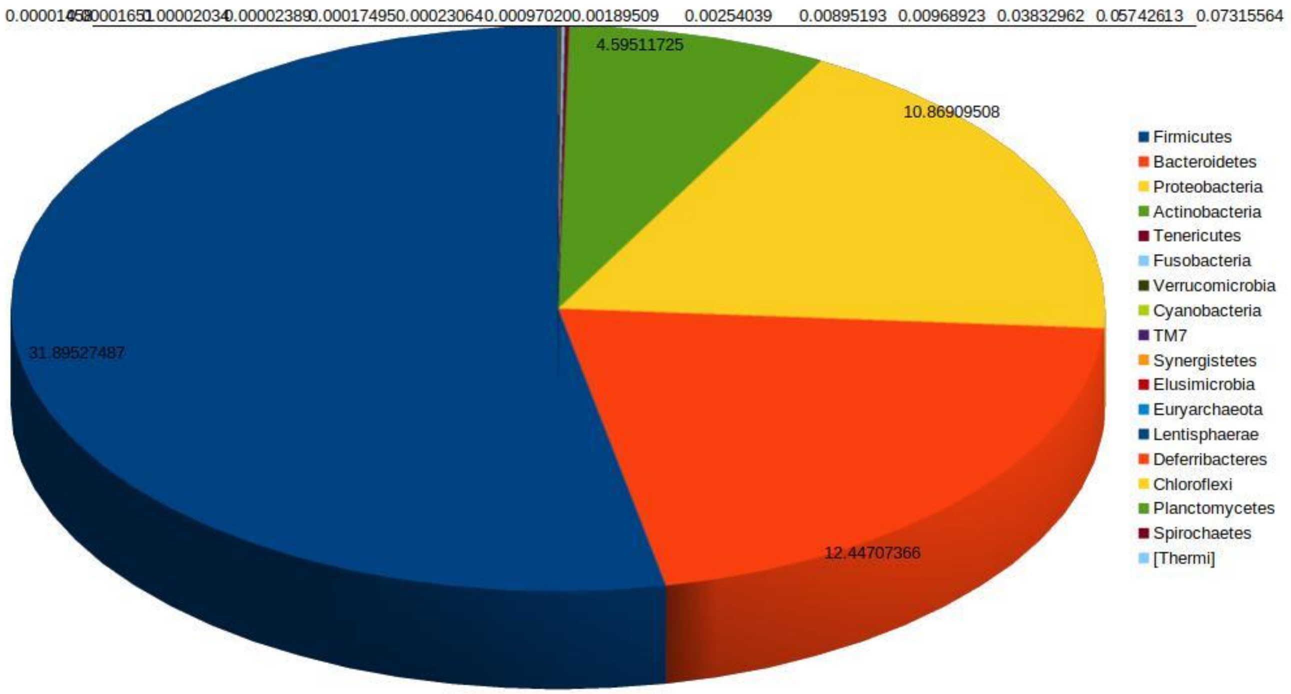

Figure S5. Pie chart showing the relative abundances of different bacterial phyla in the gut microbiota of the Trivandrum residents (N=60).

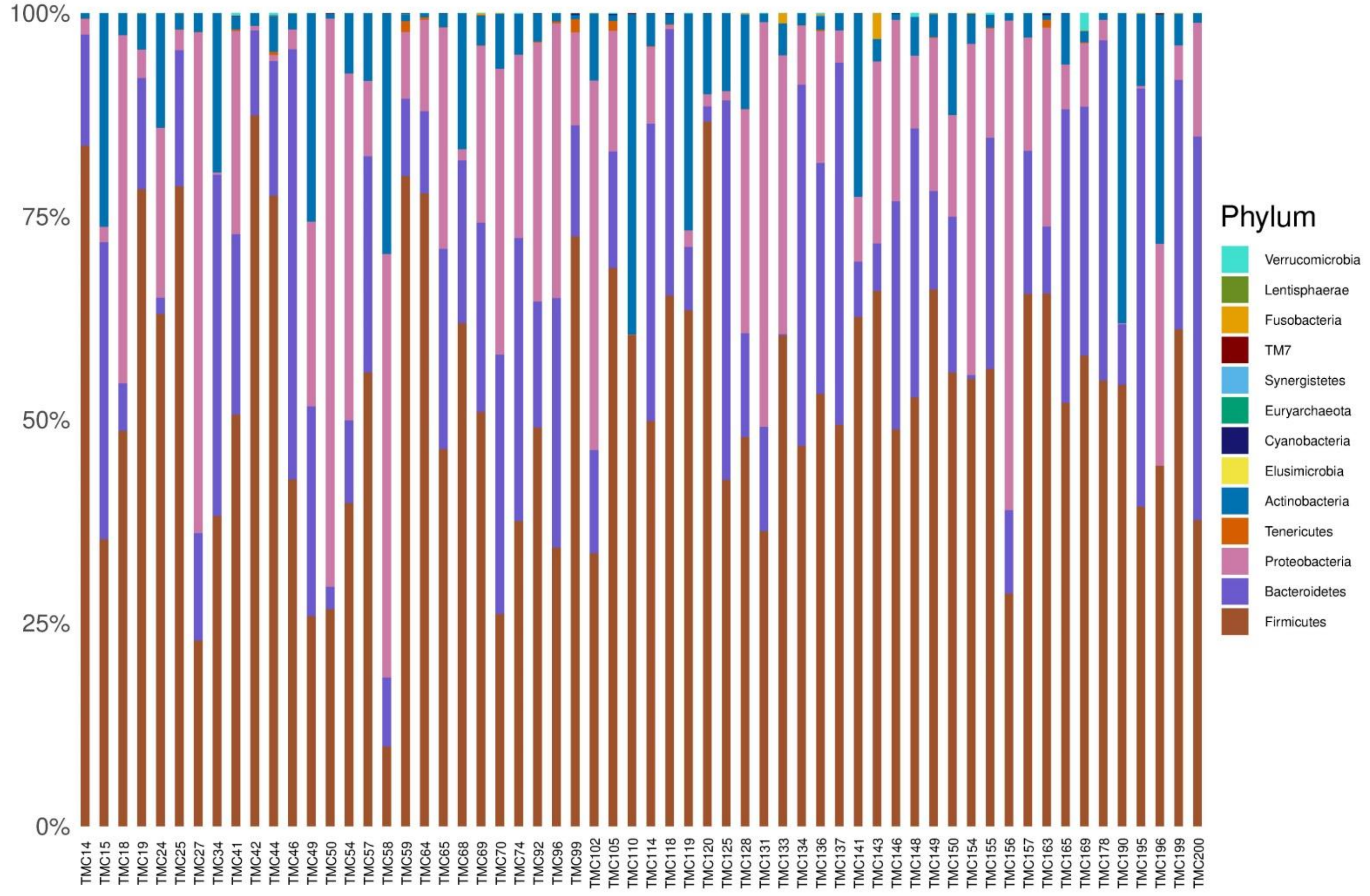

Figure S6. Analysis of gut microbiome at the phylum level for each patient. For most patients, Firmicute is the dominant phylum.

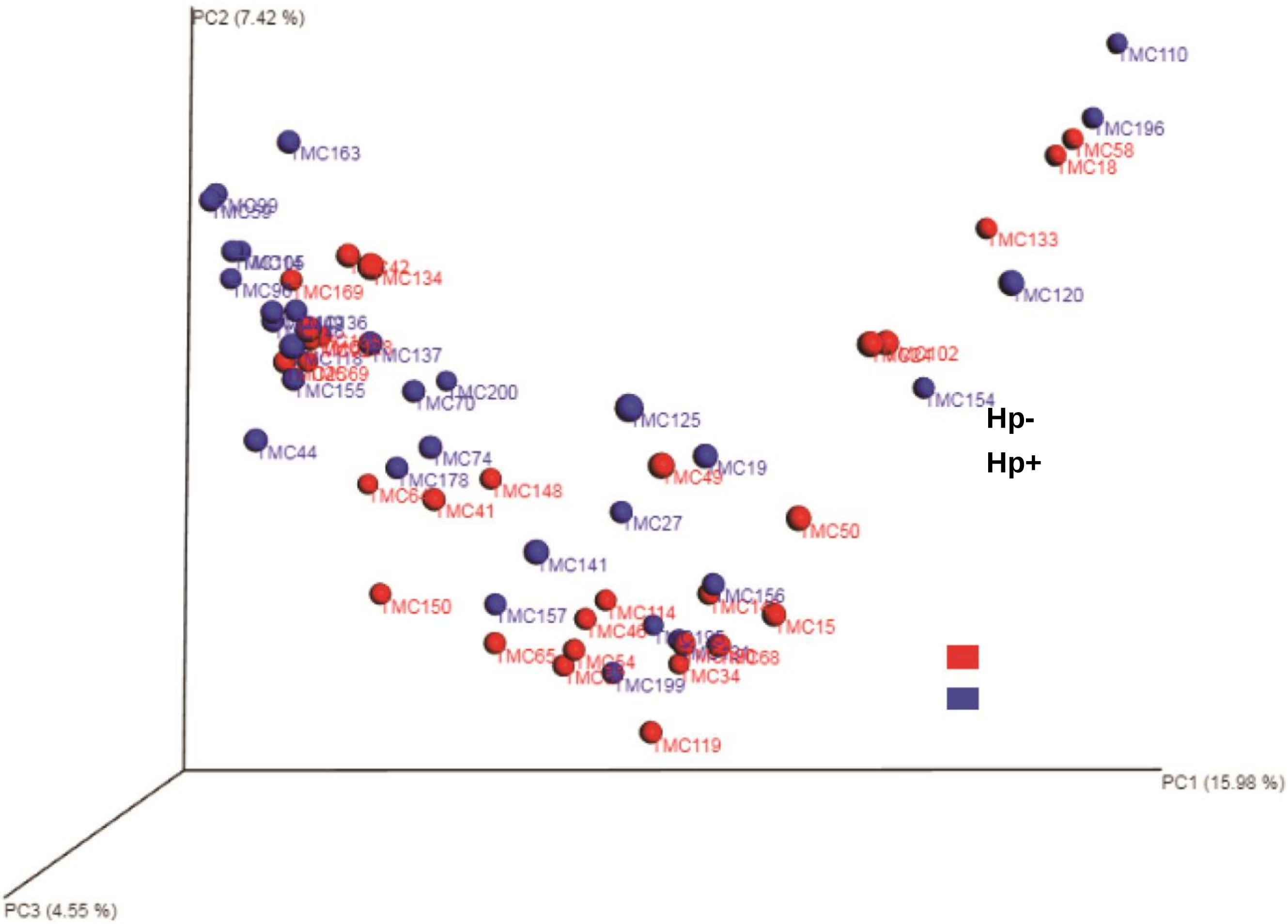

Figure S7. Principle coordinates analysis (PCoA) for the *H. pylori*+ (blue) and the *H. pylori*- (red) individuals.

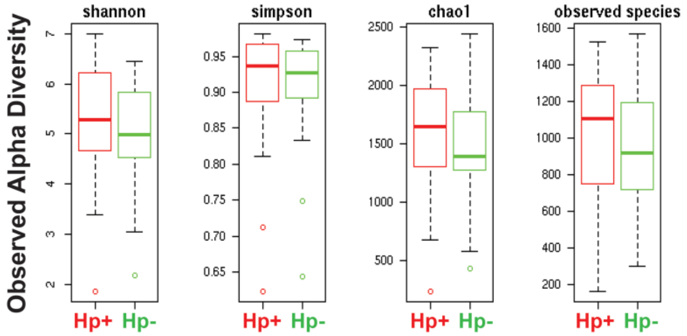

Figure S8. Observed α-diversity measured by Shannon index, Simpson index and Chao1 index analyses and the observed species for the *H. pylori*+ and the *H. pylori*- groups. In each method the *H. pylori*+ group has more diversity than the *H. pylori*- group. The reason for this higher number of microbial species in the feces of the *H pylori*+ group is unknown at present. We speculate that it could be linked to the hypochlorhydria caused by destruction of the H^+ ion secreting parietal cells due to chronic *H. pylori* colonization. Thus, it is possible that the microbial species that usually fail to tolerate low gastric pH can pass through the stomach and colonize in intestine because of the hypochlorhydric milieu in stomach created by *H. pylori* infection.

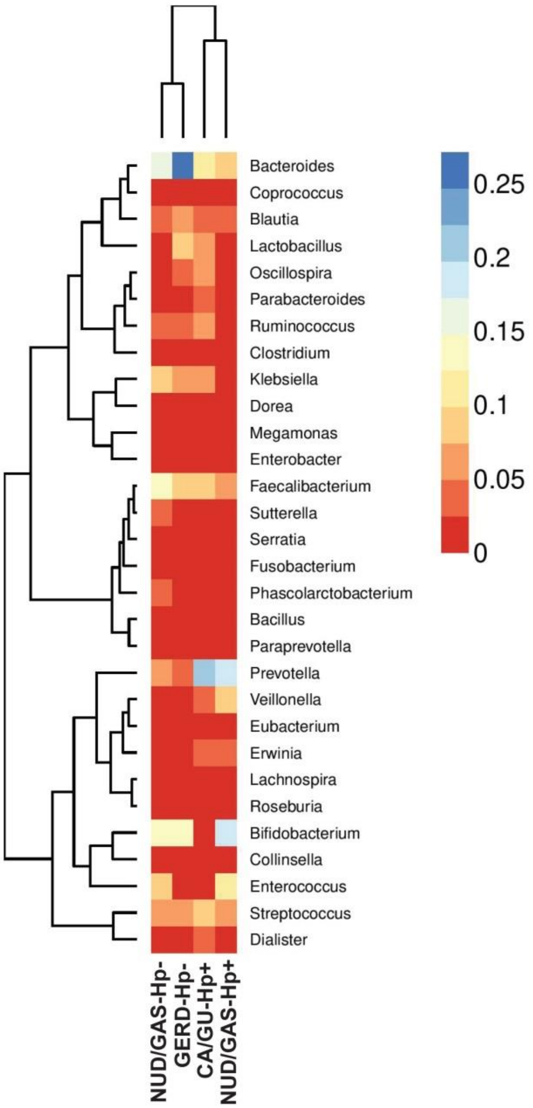

Figure S9. Heat map at the genus level for the CA/GU-Hp+, NUD/GAS-Hp+, NUD/GAS-Hp- and GERD-Hp- groups. The genus *Bifidobacterium* is remarkably low particularly in CA/GU-Hp+ group.

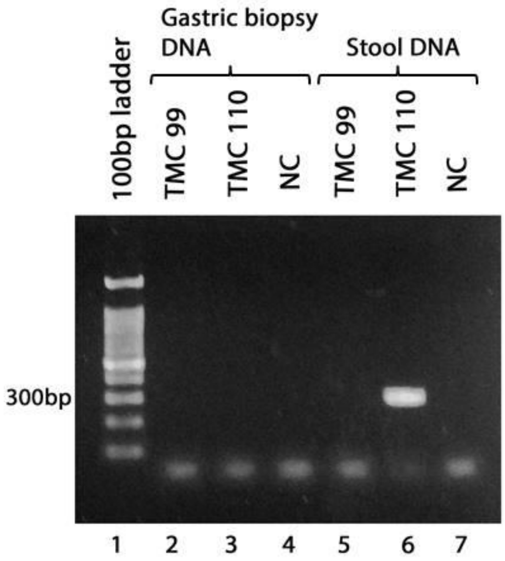

Figure S10. Gel image of *B. breve* specific PCR with gastric biopsy metagenomic DNA and stool metagenomic DNA. No amplification was observed with the gastric biopsy metagenomic DNA, while stool metagenomic DNA from TMC110 gave amplicon at 288 bp.

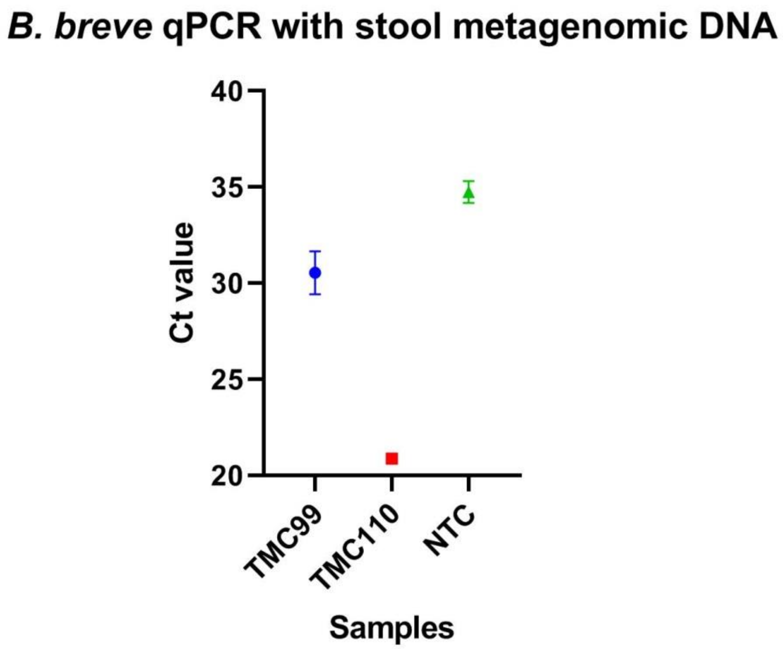

Figure S11. Graph showing the mean Ct values obtained by *B breve* qPCR with stool metagenomic DNA from TMC99 (CA/GU) and TMC110 (NUD/GAS). Higher Ct value in TMC99 indicates lower *B. breve* abundance when compared to TMC99. Error bar indicate the standard deviations.

Supplementary Tables.

Table S1. Combined data for *ureB* PCR and *H. pylori* culture for all patients.

**Total No. of samples**	***H. pylori ureB* positive**	***H. pylori* Culture positive**	**Only *ureB* positive**	**Only culture Positive**	***ureB* & Culture positive**	**Total No. of *H. pylori* positive (Culture & *ureB* PCR combined)**
**375**	**77**	**42**	**41**	**6**	**36**	**83**

Table S2. The *ureB* PCR result and the culture result for each *H. pylori*+ patient.

**S.NO**	**Sample No.**	***H. pylori ureB* pcr +ve**	***H. pylori* Culture +ve**
1	TMC 5	Positive	Positive
2	TMC 7	Positive	Positive
3	TMC 11	Positive	Negative
4	TMC 14	Positive	Negative
5	TMC 19	Positive	Positive
6	TMC 20	Positive	Positive
7	TMC 27	Positive	Positive
8	TMC 40	Positive	Positive
9	TMC 44	Positive	Positive
10	TMC 55	Positive	Negative
11	TMC 59	Positive	Positive
12	TMC 66	Positive	Negative
13	TMC 70	Negative	Positive
14	TMC 74	Positive	Positive
15	TMC 88	Negative	Positive
16	TMC 94	Positive	Positive
17	TMC 96	Positive	Positive
18	TMC 99	Positive	Positive
19	TMC 105	Positive	Negative
20	TMC 108	Positive	Negative

21	TMC 110	Negative	Positive
22	TMC 113	Positive	Negative
23	TMC 118	Positive	Positive
24	TMC 120	Positive	Positive
25	TMC 123	Positive	Negative
26	TMC 125	Positive	Positive
27	TMC 130	Positive	Negative
28	TMC 131	Positive	Negative
29	TMC 136	Positive	Positive
30	TMC 137	Positive	Negative
31	TMC 141	Positive	Positive
32	TMC 149	Positive	Positive
33	TMC 154	Positive	Positive
34	TMC 155	Positive	Positive
35	TMC 156	Positive	Negative
36	TMC 157	Positive	Negative
37	TMC 163	Positive	Positive
38	TMC 168	Positive	Positive
39	TMC 175	Positive	Negative
40	TMC 177	Positive	Negative
41	TMC 178	Positive	Negative
42	TMC 193	Positive	Negative
43	TMC 195	Positive	Negative
44	TMC 196	Positive	Negative
45	TMC 199	Negative	Positive
46	TMC 200	Positive	Negative
47	TMC 201	Positive	Negative
48	TMC 202	Positive	Negative
49	TMC 203	Positive	Negative
50	TMC 204	Positive	Negative
51	TMC 205	Positive	Negative
52	TMC 206	Positive	Positive
53	TMC 207	Positive	Negative
54	TMC 208	Positive	Negative
55	TMC 209	Positive	Negative
56	TMC 214	Positive	Negative
57	TMC 215	Positive	Positive
58	TMC 217	Positive	Negative
59	TMC 218	Positive	Negative
60	TMC 230	Positive	Positive
61	TMC 234	Positive	Positive
62	TMC 235	Positive	Positive

63	TMC 244	Positive	Negative
64	TMC 251	Positive	Negative
65	TMC 255	Positive	Negative
66	TMC 259	Positive	Negative
67	TMC 263	Positive	Positive
68	TMC 266	Positive	Negative
69	TMC 268	Positive	Positive
70	TMC 269	Positive	Positive
71	TMC 275	Positive	Positive
72	TMC 280	Positive	Positive
73	TMC 281	Positive	Negative
72	TMC 290	Positive	Positive
75	TMC 294	Positive	Positive
76	TMC 321	Negative	Positive
77	TMC 346	Positive	Negative
78	TMC 347	Positive	Negative
79	TMC 348	Positive	Negative
80	TMC 366	Positive	Positive
81	TMC 367	Negative	Positive
82	TMC 371	Positive	Positive
83	TMC 372	Positive	Negative

Table S3. Nucleotide sequences of the primers used in the study.

Target	Primer name	Sequence (5'-3')
H. pylori vacAs1/s2 for multiplex PCR	VA1-F	ATGGAAATACAACAAACACAC
	VA1-R	CTGCTTGAATGCGCCAAAC
H. pylori vacAm1/m2 for multiplex PCR	VAG-F	CAATCTGTCCAATCAAGCGAG
	VAG-R	GCGTCAAAATAATTCCAAGG
H. pylori cagA for multiplex PCR	cag5c-F	GTTGATAACGCTGTCGCTTC
	cag3c-R	GGGTTGTATGATATTTTCCATAA
H. pylori cag-empty site	Luni1	ACATTTTGGCTAAATAAACGCTG
	R5280	GGTTGCACGCATTTTCCCTTAATC
H. pylori urease B subunit	ureB-F	CGTCCGGCAATAGCTGCCATAGT
	ureB-R	GTAGGTCCTGCTACTGAAGCCTTA
H. pylori vacA mid-region for sequencing	VAm-F	GCTCATTACGGCTTCCACTAATGT
	VAm-R	GCGGTTATTGTTGTTATAAAGGGCTA
H. pylori cagA 5' end for sequencing	cagA5	GGCAATGGTGGTCCTGGAGCTAGGC
	cagA2	GGAAATCTTTAATCTCAGTTCGG
H. pylori cagA 3' end for sequenci	CAG1	ACCCTAGTCGGTAATGGGTTA
	CAG2	GTAATTGTCTAGTTTCGC

ng		
H. pylori vacAi	VacF1	GTTGGGATTGGGGGAATGCCG
	C1R	TTAATTTAACGCTGTTTGAAG
	C2R	GATCAACGCTCTGATTTGA
B. breve	BiBRE-1	CCGGATGCTCCATCACAC
	BiBRE-2	ACAAAGTGCCTTGCTCCCT
V3-V4 region of bacterial 16S rRNA gene	16S V3-V4 F	TCGTCGGCAGCGTCAGATGTGTATAAGAGACAGCCTACGGGNGGCWGCAG
	16S V3-V4 R	GTCTCGTGGGCTCGGAGATGTGTATAAGAGACAGGACTACHVGGGTATCTAATCC

Table S4. The incidence of different upper GI diseases in Kerala and Sikkim.

Clinical Status	Total number	Percentage of total incidence of upper GI diseases in our study	Percentage of total incidence of upper GI diseases in Sikkim
**Gastric cancer (CA)***	23	6.1%	1.2%
**Gastric ulcer (GU)***	22	5.9%	1.4%
**Duodenal ulcer (DU)***	6	1.6%	4.4%
**Gastritis (GAS)**	135	36%	51.07%
**Non-ulcer dyspepsia (NUD)**	97	25.9%	17%
**Gastroesophageal reflux disease (GERD)**	92	24.5%	11.5%

Footnote: The severe gastric diseases like gastric cancer or peptic ulcer (gastric and duodenal ulcers) are shown with *.

Table S5. Genotypes of *H. pylori* strains isolated from Trivandrum

***H. pylori* Genotype**	**Number of Strains(n=42)**	**% of strains**
vacAs1	39	92.9
vacAs2	3	7.1
vacAm1	32	76.2
vacAm2	10	23.8
vacAi1	37	88.1
vacAi2	5	11.9
cagA(+)	38	90.5
cagA(-)	4	9.5
cag-PAI(-)	4	9.5
vacAs1i1m1cagA(+)	31	73.8
vacAs1i1m2cagA(+)	5	11.9
vacAs1i2m2cagA(+)	2	4.8
vacAs2i2m2cagA(-)	3	7.1
vacAs1i1m1cagA(-)	1	2.4

Table S6. The CagA C-terminal end variants and their association with different clinical status.

CagA types	PUD (GU +DU)		Gastric cancer		Gastritis		NUD		GERD	
	N	%	N	%	N	%	N	%	N	%
A B-C (28)	3	10.7	3	10.7	11	39.3	6	21.4	5	17.9
A B-C-C (7)					5	71.4	1	14.3	1	14.3
A B (2)					1	50	1	50		
A-C-C (1)							1	100		

Table S7. *H. pylori* genotypes and clinical status.

H. pylori genotypes	**CA/PUD (N=6)**	**NUD/GAS (N=29)**	**GERD (N=7)**
vacAs1i1m1cagA(+)	3 (50%)	23 (79.3%)	5 (71.4%)
vacAs1i1m2cagA(+)	1 (16.7%)	3 (10.3%)	1 (14.3%)
vacAs1i2m2cagA(+)	2 (33.3%)		
vacAs2i2m2cagA(-)		2 (7%)	1 (14.3%)
vacAs1i1m1cagA(-)		1 (3.4%)	

Table S8. Details of the 60 patients (30 *H. pylori*+ and 30 *H. pylori*-) included in gut microbiome analysis.

***H. pylori* +**	**30**	***H. pylori* -**	**30**
Number of Male	14	Number of Male	14
Number of Female	16	Number of Female	16
Ave Age	50.5	Ave Age	50.4

Table S9. The gut microbial diversity for each *H. pylori*+ patients vs the *H. pylori*- patients.

Hp+	Hp-

Sample No.	Age range (years)	Shannon	Simpson	Chao1	Observed OTU	Sample No.	Age range (years)	Shannon	Simpson	Chao1	Observed OTU
TMC 14	60-65	156886	90651	70773	1508	TMC 54	60-65	179814	136796	127716	896
TMC 19	45-50	67092	39655	38257	642	TMC 134	45-50	144923	98742	95625	937
TMC 27	42-47	142615	103431	97622	877	TMC 15	42-47	135463	85220	82632	669
TMC 44	50-55	177433	111079	103770	1389	TMC 119	50-55	130451	90034	87867	847
TMC 59	40-45	177433	111079	103770	1249	TMC 148	40-45	304907	191067	185366	1219
TMC 70	37-42	274865	194727	178565	1153	TMC 25	37-42	115287	81595	77844	1196
TMC 74	58-63	169567	113453	107825	1014	TMC 34	58-63	226229	142514	133470	909
TMC96	47-53	274481	199154	181589	1357	TMC 133	47-53	157202	111505	106505	526
TMC 99	40-45	214944	134907	122870	1524	TMC 128	40-45	90688	41669	40459	367
TMC 105	48-53	105253	75109	67132	1319	TMC 69	48-53	207598	138245	127521	1568
TMC 110	40-45	126278	80305	76850	163	TMC 114	40-45	205644	137189	132542	1120
TMC 118	37-43	187981	113335	109469	1180	TMC 68	37-43	155775	101596	97717	852
TMC 120	43-48	90688	41669	40459	367	TMC 42	43-48	142935	89869	87385	852
TMC 125	62-67	99034	63517	61923	708	TMC 46	62-67	125313	74374	70715	877
TMC 131	42-47	158425	104898	100753	753	TMC 41	42-47	190599	129248	125204	1202
TMC 136	65-70	143676	90727	85838	1229	TMC 18	65-70	118265	77099	73267	297
TMC 137	55-60	191513	105505	98498	1040	TMC 150	55-60	330326	228543	219723	1367
TMC 141	37-42	230459	154378	151638	1032	TMC 58	37-42	135164	95478	92874	342
TMC 146	62-67	218884	153045	143737	1304	TMC 169	62-67	96071	65014	62274	984
TMC 149	30-35	162523	100999	96005	1346	TMC 49	30-35	112148	65775	61745	705
TMC 154	45-50	149888	95679	90262	584	TMC 24	45-50	68500	39969	38847	546
TMC 155	32-37	318019	211074	197213	1222	TMC 102	32-37	97953	60182	58757	599
TMC 156	50-55	170135	125055	118496	745	TMC 65	50-55	239837	174250	164726	1170
TMC 157	43-48	205892	148491	144984	1291	TMC 57	43-48	137899	98346	91520	989
TMC 163	65-70	105916	61067	57805	1035	TMC 92	65-70	220830	150421	145833	1284
TMC 178	60-65	205219	142948	131838	1189	TMC 50	60-65	147439	100604	98351	717

TMC195	65-70	182027	123675	114366	857	TMC143	65-70	353175	245998	226663	936
TMC196	42-47	261328	177387	169514	461	TMC190	42-47	209931	143424	134883	792
TMC199	50-55	151610	96902	90876	1144	TMC165	50-55	126044	83400	77656	958
TMC200	42-47	118088	76502	68590	1075	TMC64	42-47	155775	101596	97717	852
Total		5238152	3440403	3221287	30757	Total		5062185	3379762	3223404	26575

Table S10. Metadata of the patients included in the 16S rRNA gene sequence analysis among 4 groups- CA/GU-Hp+, NUD/GAS-Hp+, NUD/GAS-Hp-, GERD-Hp-.

**Group name**	**Sample ID**	**Clinical status**	**Age range (years)**	**Hp+/Hp-**
CA(Hp+)/GU(Hp+)	TMC14	Gastric cancer	62-67	Hp+
	TMC99	Gastric cancer	40-45	Hp+
	TMC178	Gastric cancer	62-67	Hp+
	TMC200	Gastric cancer	45-50	Hp+
	TMC146	Gastric ulcer	60-65	Hp+
	TMC154	Gastric cancer	42-47	Hp+
	TMC163	Gastric ulcer	65-70	Hp+
NUD(Hp+)/Gastritis(Hp+)	TMC125	NUD	62-67	Hp+
	TMC110	NUD	40-45	Hp+
	TMC199	Gastritis	50-55	Hp+
	TMC19	NUD	45-50	Hp+
	TMC74	NUD	60-65	Hp+
	TMC196	Gastritis	42-47	Hp+
	TMC137	NUD	55-60	Hp+
NUD(Hp-)/Gastritis(Hp-)	TMC54	Gastritis	60-65	Hp-
	TMC128	NUD	40-45	Hp-
	TMC92	Gastritis	65-70	Hp-
	TMC143	Gastritis	62-67	Hp-
	TMC58	NUD	37-42	Hp-
	TMC18	NUD	65-70	Hp-
	TMC65	Gastritis	50-55	Hp-
GERD(Hp-)	TMC34	GERD	60-65	Hp-
	TMC41	GERD	40-45	Hp-
	TMC24	GERD	47-53	Hp-
	TMC69	GERD	47-53	Hp-
	TMC57	GERD	45-50	Hp-
	TMC15	GERD	42-47	Hp-
	TMC42	GERD	43-48	Hp-

Table S11. Metadata of the age and sex matched patients (N=6 and 3 pairs) included in the fecal shotgun metagenomic sequencing analysis.

Pairs	Group name	Sample ID	Clinical status	Age range (years)	Hp+/Hp-
Pair 1	CA(Hp+)/GU(Hp+)	TMC99	Gastric cancer	40-45	Hp+
	NUD(Hp+)/Gastritis(Hp+)	TMC110	NUD	40-45	Hp+
Pair 2	CA(Hp+)/GU(Hp+)	TMC163	Gastric ulcer	65-70	Hp+
	NUD(Hp+)/Gastritis(Hp+)	TMC125	NUD	65-70	Hp+
Pair 3	CA(Hp+)/GU(Hp+)	TMC154	Gastric cancer	42-47	Hp+
	NUD(Hp+)/Gastritis(Hp+)	TMC120	NUD	42-47	Hp+

Table S12. The Nanopore read statistics for each sample.

Read Statistics	TMC99	TMC110	TMC_163	TMC_125	TMC_154	TMC_120
Reads Generated :	6,69,400	1,41,339	2,12,156	1,57,603	1,98,330	2,84,964
Maximum Read Length :	16575	24116	16443	18319	19332	19654
Minimum Read Length :	133	87	1	1	1	1
Average Read Length :	1339.7	1321.1	1412.4	1506.2	1128.2	1158.9
Median Read Length :	1633	286	3407.5	218	2443	1678
Total Read Length :	89,68,17,108	18,67,26,069	29,96,45,341	23,73,78,327	22,37,53,693	33,02,39,186
Total Number of Non-ATGC Characters :	0	0	0	0	0	0
Percentage of Non-ATGC Characters :	0	0	0	0	0	0
Reads >= 100 bp :	6,69,400	1,41,336	2,10,389	1,55,726	1,94,039	2,80,740
Reads >= 200 bp :	6,67,500	1,40,382	2,01,646	1,44,708	1,68,587	2,60,463
Reads >= 500 bp :	5,99,414	77760	1,45,287	99245	1,06,778	1,82,551
Reads >= 1 Kbp :	3,83,236	44816	99602	67585	65321	1,09,835
Reads >= 10 Kbp :	88	1062	146	673	663	376
Reads >= 1 Mbp :	0	0	0	0	0	0
N50 value :	1660	2938	2408	3066	2244	1872

Table S13. Abundance of each domain present in each sample as discerned by Nanopore sequencing.

Domain	TMC110	TMC120	TMC125	TMC154	TMC163	TMC99
Bacteria	50479	51993	51855	51796	51492	51044
Archaea	0	0	0	0	49	302
Eukaryota	1250	70	176	165	196	289
Viruses	0	0	0	0	124	81

Table S14. Mean Ct values for gastric biopsy and stool DNA for TMC99 (CA/GU) and TMC110 (NUD/GAS).

**Template source**	Metagenomic Biopsy DNA		Metagenomic Stool DNA		
**Sample**	TMC99	TMC110	TMC99	TMC110	NTC
**Mean Ct value**	Undetermined	34.189	30.378	20.893	34.736

NTC: No-template Control. CA: Gastric cancer; GU: Gastric ulcer; NUD: Non-ulcer dyspepsia; GAS: Gastritis
